# Preclinical pharmacodynamic evaluation of drug candidate SKLB-178 in the treatment of non-small cell lung cancer

**DOI:** 10.18632/oncotarget.14597

**Published:** 2017-01-11

**Authors:** Lei Zhong, Jiao Yang, Zhixing Cao, Xin Chen, Yiguo Hu, Linli Li, Shengyong Yang

**Affiliations:** ^1^ State Key Laboratory of Biotherapy and Cancer Center, West China Hospital, West China Medical School, Sichuan University/Collaborative Innovation Center of Biotherapy, Sichuan 610041, China; ^2^ Personalized Drug Therapy Key Laboratory of Sichuan Province, Hospital of the University of Electronic Science and Technology of China and Sichuan Provincial People's Hospital, Sichuan 610072, China; ^3^ Pharmacy College, Chengdu University of Traditional Chinese Medicine, Sichuan 611137, China; ^4^ Key Laboratory of Drug Targeting and Drug Delivery System, Ministry of Education, West China School of pharmacy, Sichuan University, Sichuan 610041, China

**Keywords:** SKLB-178, non-small cell lung cancer, multikinase inhibitor, EGFR, Src

## Abstract

Non-small cell lung cancer (NSCLC) is a serious life-threatening malignancy. Epidermal growth factor receptor (EGFR) tyrosine kinase inhibitors, such as Gefitinib and Erlotinib, are effective clinical medicines for advanced NSCLC patients harboring EGFR-activating mutations. However, this therapy just benefits a small percentage of sufferers. Worse still, all patients treated with drugs ultimately develop resistance. Hence, there is still an unmet medical need among patients with NSCLC. In this account, we report a novel multikinase inhibitor SKLB-178, which potently inhibits both EGFR-activating and resistant mutations, as well as the activities of Src and VEGFR2 kinases. SKLB-178 potently inhibited cancer cell growth in both Gefitinib-sensitive and resistant NSCLC cells. Meanwhile, SKLB-178 significantly suppressed the migration, invasion and tube formation of endothelial cells, and the growth of intersegmental vessel in zebrafish. The *in vivo* pharmacodynamic studies further demonstrated that SKLB-178 had wider potency than Gefitinib, and could significantly prolong survival of animals in A549 experimental metastasis model. These advantages together with the low toxicity of SKLB-178 indicate that SKLB-178 deserves to be further developed as a potential drug candidate for NSCLC therapy.

## INTRODUCTION

Lung cancer is the leading cause of cancer-related mortality worldwide. Non-small cell lung cancer (NSCLC) accounts for approximately 85% of all lung cancer cases. Molecular targeted therapy is considered a more promising strategy for the treatment of advanced NSCLC compared with cytotoxic chemotherapy, given its relatively high efficiency and weak side effect [1, 2]. Gefitinib, a first generation small-molecule epidermal growth factor receptor (EGFR) inhibitor, is highly active in NSCLC patients harboring EGFR-activating mutations including exon 19 deletion (E746-A750) mutation, exon 21 L858R mutation, and EGFR amplification [3–5]. It has successfully launched a new era of NSCLC therapy. Nevertheless, this therapy just benefits a sub set of NSCLC patients [6]. Furthermore, all sufferers treated with drugs ultimately develop resistance [7–9]. These disadvantages seriously restrict the efficacy of EGFR inhibitors in clinic. Therefore, development of novel anti-NSCLC medicines with broad activity and capability to overcome Gefitinib resistance is still strongly demanded.

NSCLC is a highly heterogeneous disease with multi-factorial pathological signatures. There is multiple cross-stimulation among targets along several pathways of signal transduction that result in malignancy of NSCLC. Therefore, agents targeting multiple tumor-associated pathways may be expected to benefit the clinical therapeutic effect of NSCLC [10, 11]. Among targets associated with NSCLC, EGFR is a well characterized mutated oncogene, and undisputed the preferred therapeutic target [3, 12]. Moreover, the non-receptor tyrosine kinase Src is a promising target for cancer therapy, as inhibition of Src leads to suppression of multiple oncogenic pathways including PI3K/Akt, Ras/Raf/MAPK, JAK/Stats and SFK/FAK/p130CAS cascades [13, 14]. Numerous studies have shown that aberrant Src activation is implicated in the development, metastasis and even resistance of NSCLC [15–19]. Src inhibitors could improve the efficacy of some molecular targeted drugs and cytotoxic agents for NSCLC therapy in preclinical studies [20, 21]. Hence, inhibition of Src activity may be of particular clinical interest in the treatment of NSCLC. In addition, accumulating evidence from the literature indicates that anti-angiogenic therapy is a well-established and effective strategy for the treatment of solid tumors [22–24]. NSCLC is also a highly vascular tumor, and numerous angiogenesis inhibitors have been tested clinically for NSCLC therapy [25–28]. Thus, targeting angiogenesis- associated targets, especially VEGFR2 which plays a pivotal role in pathological angiogenesis, is also a particularly attractive therapeutic approach for NSCLC.

In this account, we report a novel multikinase inhibitor SKLB-178, which efficaciously inhibits both EGFR-activating and resistant mutations, as well as the activities of Src and VEGFR2 kinases. SKLB-178 potently inhibited NSCLC cell proliferation and induced apoptosis in cellular assays. Meanwhile, SKLB-178 could significantly suppress the migration, invasion and tube formation of endothelial cells, and the growth of intersegmental blood vessel in zebrafish. The *in vivo* pharmacodynamic studies further demonstrated that SKLB-178 had wider potency compared with Gefitinib, and could exert its anti-NSCLC activity directly by suppression of tumor cell growth and indirectly by inhibiting angiogenesis. Furthermore, treatment with SKLB-178 led to a significant improvement in survival in A549 experimental metastasis model. These advantages together with the low toxicity of SKLB-178 indicate that SKLB-178 is a promising drug candidate for NSCLC therapy.

## RESULTS

### Kinase inhibition activity of SKLB-178

The kinase inhibition potency of SKLB-178 was measured using the gold-standard ^33^P radiolabeled technology. As shown in Supplementary Table S1, SKLB-178 completely inhibited the activities of EGFR, EGFR L858R mutation, EGFR L858R/T790M mutation, KDR (VEGFR2), and Src either at 10 μM or at a lower dose of 1 μM. SKLB-178 also showed inhibitory activity against MET kinase at 10 μM (88% inhibition rate), and ErbB4 at 1 μM (100% inhibition rate). Additionally, SKLB-178 exhibited a relatively low inhibitory rate of 10%, 0%, 1%, 0%, 0%, 27%, 2%, and 0% against FAK, FGFR1, MEK, MKK4, mTOR, PDGFRβ, PI3K, and PLK1, respectively, at 10 μM. Similarly, SKLB-178 just inhibited the activities of IGF-1R, PDGFRα, and Syk by 11%, 58%, and 28%, respectively, at 1 μM. Taken together, these results indicated that SKLB-178 was a multikinase inhibitor mainly targeting EGFR mutations, Src, and VEGFR2.

### SKLB-178 inhibits NSCLC cell viability and proliferation *in vitro*

**Figure 1:**
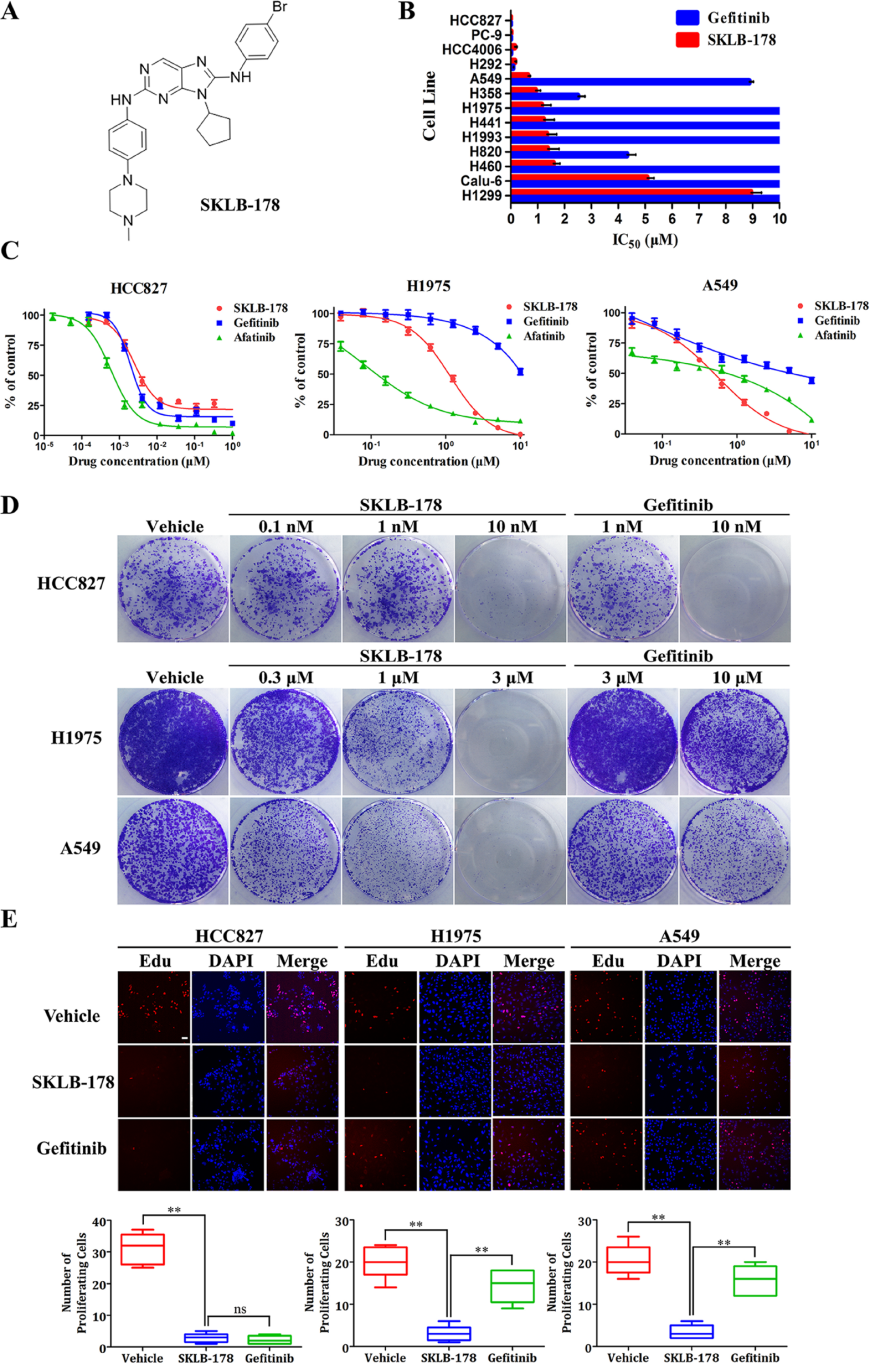
SKLB-178 treatment inhibits the growth of a subset of NSCLC cell lines (**A**) The chemical structure of SKLB-178. (**B**) Average IC_50_ values of SKLB-178 and Gefitinib for 13 indicated NSCLC cell lines. The columns without error bars indicate the insensitive cell lines (IC_50_ values >10 μM). Column, mean; bars, SD. (**C**) Anti-viability assay of SKLB-178, Gefitinib and Afatinib against three typical NSCLC cells HCC827, H1975 and A549. Points, mean values; bars, SD. (**D**) Long-term colony formation assay of HCC827, H1975 and A549 cells. (**E**) The fluorescence microscopic appearance of EdU and DAPI on three typical NSCLC cells after treatment with 100 nM (for HCC827) or 3 μM (for H1975 and A549) SKLB-178 and the same concentration of Gefitinib. EDU-positive cells (red) are quantified for statistics. Scale bar represent 50 μm. Horizontal bars, means (*n* = 6). ***P < 0.001; ns, no significant difference.

The anti-viability activity of SKLB-178 was tested in a panel of 13 NSCLC cell lines using MTT assay. SKLB-178 could potently inhibit the viability of HCC827, PC-9, HCC4006, H292, A549, H358, H1975, H441, H1993, H820, and H460, with IC_50_ values of less than 2 μM, exhibiting a wider range of activity than Gefitinib (Figure 1B). Three typical NSCLC cell lines HCC827 (EGFR^delE746-A750^), H1975 (EGFR^L858R/T790M^) and A549 (KRAS^G12S^) were selected for further comparative studies. As denoted in Figure 1C, SKLB-178 showed comparable anti-viability activity compared with Gefitinib on Gefitinib-sensitive HCC827 cells, the corresponding IC_50_ values for SKLB-178 and Gefitinib were 2.5 nM and 1.6 nM, respectively. Meanwhile, SKLB-178 efficiently inhibited the viability of Gefitinib-resistant H1975 and A549 cells, with IC_50_ values of 1.1 μM and 0.58 μM, respectively. As a positive control for EGFR gatekeeper mutation, the covalent inhibitor Afatinib exhibited strong inhibitory effects on EGFR-mutant cells, but showed weaker anti-viability activity than SKLB-178 on A549 cells.

To complement the results from short-term treatments, long-term colony formation assay was then performed to assess the cytoreductive activity of SKLB-178. As shown in Figure 1D, SKLB-178 dose-dependently decreased the formation of colonies in all of the three NSCLC cell lines, while Gefitinib significantly reduced colonies of HCC827 cell line. The similar results were also observed in EdU cell proliferation assay. Both SKLB-178 and Gefitinib markedly reduced the number of proliferating HCC827 cells (red nuclei), but only SKLB-178 had significant anti-proliferation effects on H1975 and A549 cells (Figure 1E). To sum up, these data indicated that SKLB-178 had efficacies of anti-viability and anti-proliferation on both Gefitinib-sensitive and resistant NSCLC cells, showing a wider potency than Gefitinib.

### SKLB-178 induces G_0_-G_1_ phase arrest and apoptosis in NSCLC cells

**Figure 2:**
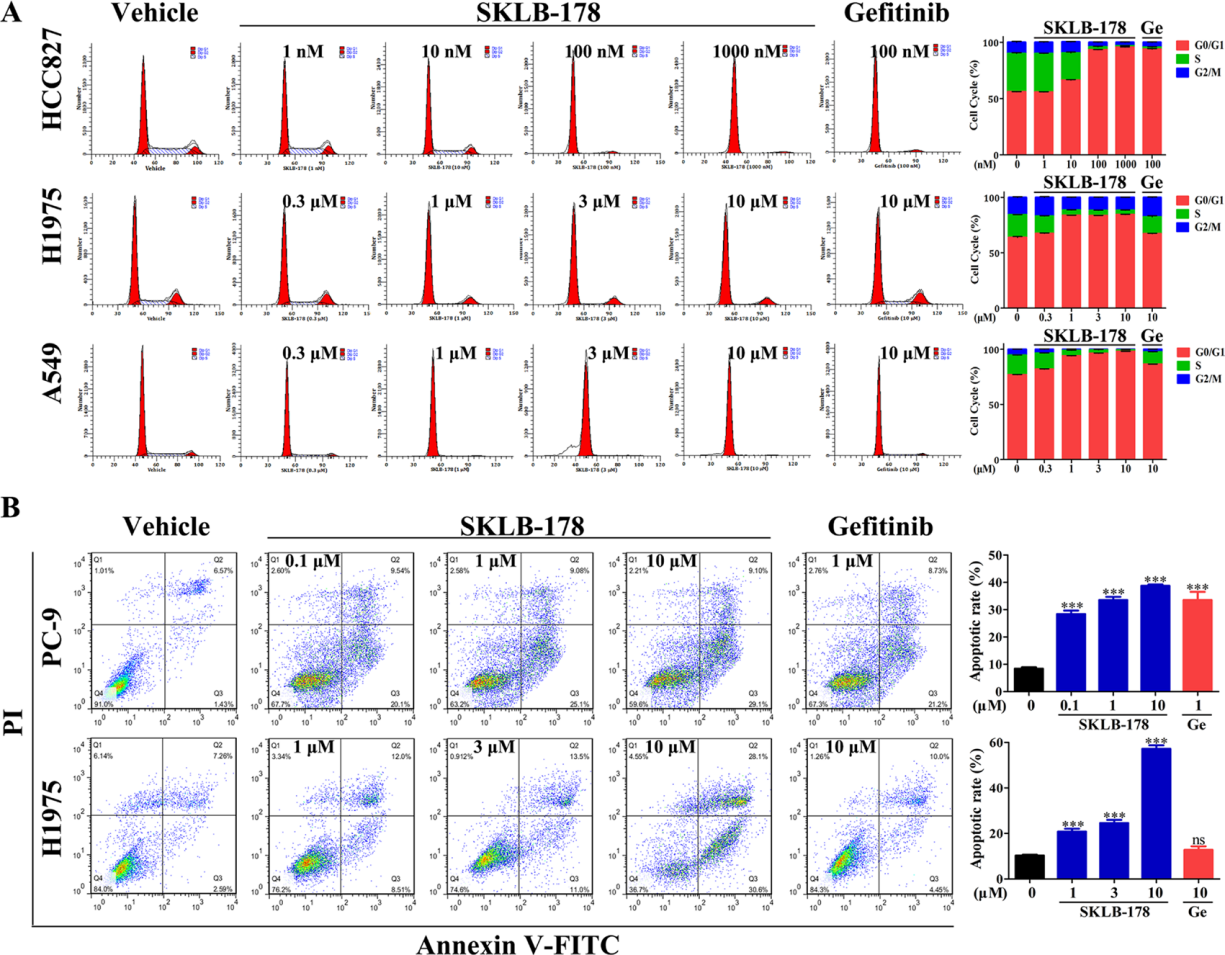
SKLB-178 induces G_0_/G_1_ phase arrest and apoptosis in NSCLC cells (**A**) Influence of SKLB-178 and Gefitinib on cell cycle progression in NSCLC cells. Representative images for three NSCLC cell lines treated with indicated concentrations of SKLB-178 or Gefitinib are shown on the left, and the percentages of the cell cycle are also presented on the right. Data shown in the histogram are means ± SD from three independent experiments. (**B**) PC-9 and H1975 were cultured in the presence of SKLB-178, Gefitinib or vehicle, followed by apoptosis detection using Annexin V/PI co-staining. The assays were performed in triplicate, and the representative images are shown in the graph (left). The percentage of Annexin V-positive cells is quantified for apoptotic rate statistics. ***P < 0.001 *vs* vehicle; ns, no significant difference.

We performed flow cytometry of SKLB-178-exposed cells to determine its role on cell cycle progression and apoptosis. In HCC827 cells, SKLB-178 significantly induced cell-cycle arrest in G_0_/G_1_ phase at concentrations higher than 10 nM (Figure 2A). Similar results were obtained after treatment with Gefitinib, and the percentage of cells in G_0_/G_1_ phase was increased from 56.02% to 93.42% and 93.81% for SKLB-178 (100 nM) and Gefitinib (100 nM), respectively. In H1975 and A549 cells, SKLB-178 also led to accumulation of cells in G_0_/G_1_ phase in a dose-dependent manner, however, Gefitinib at the concentration of 10 μM just had a slight effect on cell-cycle arrest (Figure 2A).

The NSCLC cells PC-9 (harboring the same EGFR-activating mutation as HCC827) and H1975 were used for cell apoptosis analysis. Exposure of both cells to SKLB-178 for 24 h resulted in obvious increases of Annexin V-positive populations in a concentration-dependent manner (Figure 2B), demonstrating that SKLB-178 had strong effect on inducing apoptosis. By contrast, Gefitinib could also induce apoptosis in PC-9 cells at a concentration of 1 μM, however, the apoptotic rate of Gefitinib-treated H1975 cells showed no significant difference compared with vehicle (Figure 2B). Taken together, SKLB-178 could effectively induce cell-cycle arrest and apoptosis in NSCLC cells, no matter they are sensitive or resistant to Gefitinib.

### Targeting of multiple signaling pathways by SKLB-178 in NSCLC cells

**Figure 3:**
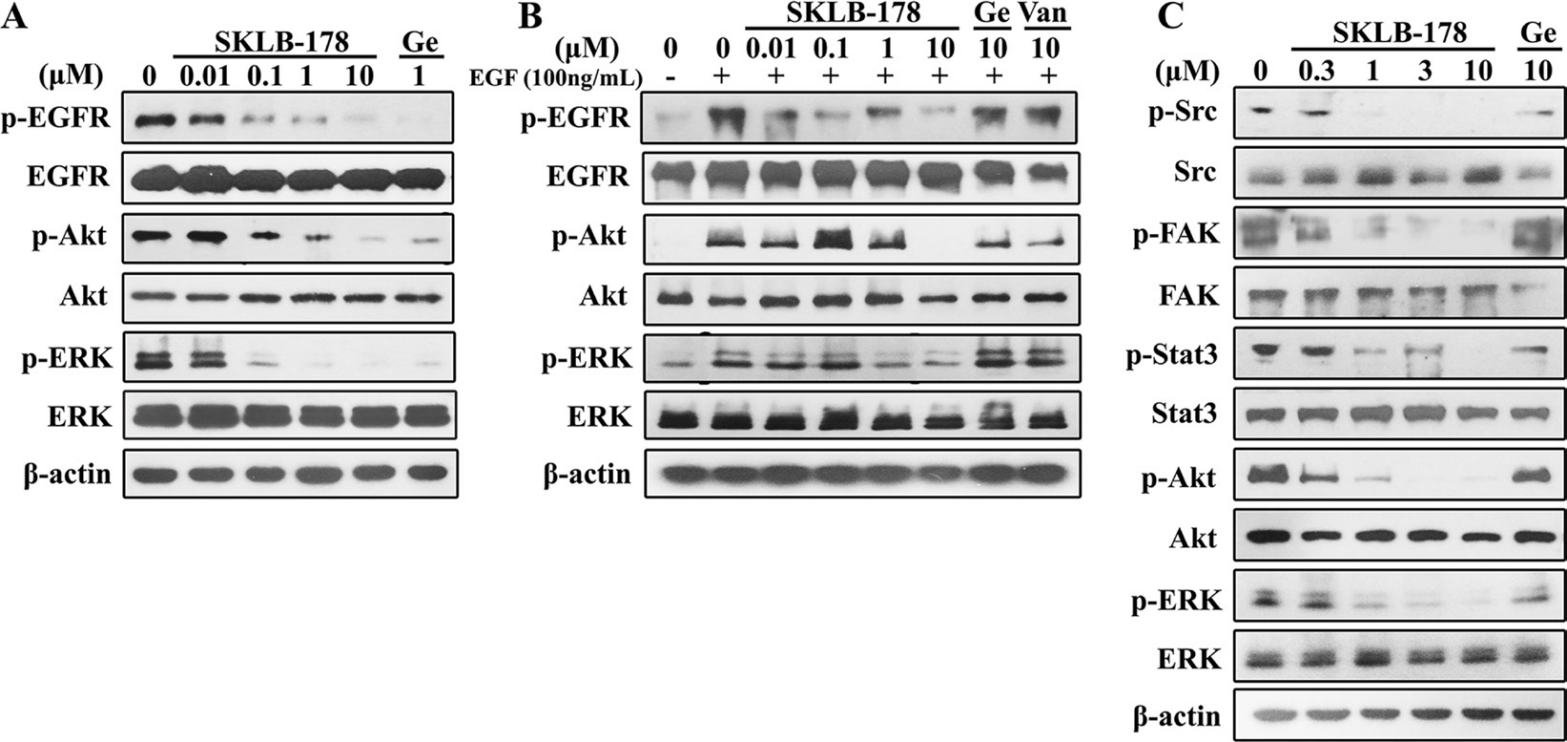
Inhibition of EGFR and Src autophosphorylation and inactivation of downstream signaling proteins in NSCLC cells by SKLB-178 (**A**) HCC827 cells were treated with vehicle, 1 μM Gefitinib, or serial dilutions of SKLB-178 for 8 h. Protein extracts were then separated for western blot analysis. (**B**) Serum-starved H1975 cells were treated with vehicle, SKLB-178, Gefitinib or Vandetanib for 2 h, followed by stimulating with 100 ng/mL EGF for 15 min. Cells were then lysed for western blot assay. (**C**) A549 cells were treated with indicated agents for 24 h. Then cells were lysed and the proteins were analyzed by immunoblot.

Western blot analysis was performed to assess the ability of SKLB-178 in the blockade of EGFR and Src signaling pathways. In HCC827 cells, SKLB-178 potently inhibited phosphorylation of EGFR, and the downstream signaling proteins Akt and ERK at concentrations higher than 0.1 μM (Figure 3A). Similar effects were also observed in cells treated by Gefitinib (1 μM). In the EGF-stimulated H1975 cells, SKLB-178 restrained EGF-dependent phosphorylation of EGFR, Akt, and ERK in a dose-dependent manner, with an estimated IC_50_ value of 1 μM (Figure 3B). In contrast, the EGFR signaling in H1975 cells was not significantly inhibited by either Gefitinib or Vandetanib at the concentration of 10 μM (Figure 3B). The inhibitory activity of SKLB-178 against Src and its downstream pivotal proteins was determined in A549 cells. As shown in Figure 3C, Src phosphorylation was significantly blocked at concentrations higher than 1 μM. The downtream kinases including FAK, Stat3, Akt, and ERK were also dose-dependently inhibited with IC_50_ values between 0.3 and 1 μM, while Gefitinib had little inhibition effect against Src cascades even at 10 μM concentration. Collectively, these results suggested that SKLB-178 was a multikinase inhibitor, and inhibited the growth of NSCLC cells mainly through suppression of EGFR and Src signalings.

### Anti-angiogenesis effect of SKLB-178

**Figure 4:**
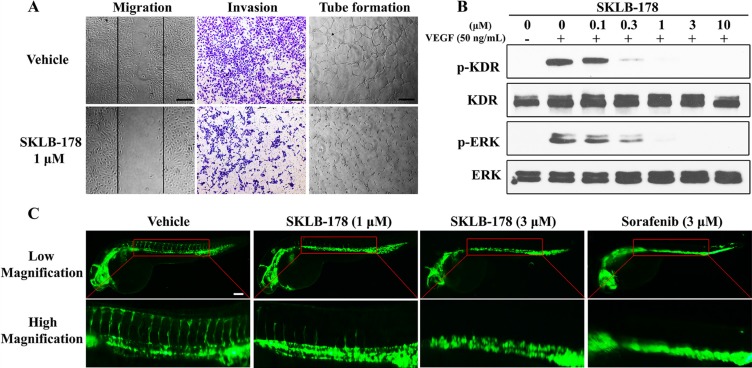
SKLB-178 inhibits angiogenesis through blockade of VEGFR signaling pathway (**A**) SKLB-178 inhibited migration, invasion, and tube formation of endothelial cells. The concentration of SKLB-178 used in the HUVECs assay were all 1 μM. The index lines in the migration assay indicate the original scratch position. Scale bars represent 100 μm. (**B**) SKLB-178 inhibited the phosphorylation of VEGFR and its downstream signaling ERK in VEGF-stimulated HUVECs. HUVECs were serum starved overnight, and then incubated with vehicle or serial dilutions of SKLB-178 for 2 h, followed by treatment with 50 ng/mL VEGF for 15 min. Cells were then lysed for western blot assay. (**C**) SKLB-178 inhibited zebrafish embryonic angiogenesis. Fluorescent images were taken after treatment with indicated agents. Scale bar represents 100 μm.

Angiogenesis arising from endothelial cells is a complex process, in which endothelial cells migration, invasion and tube formation are all pivotal steps. We carried out wound healing assay to evaluate the inhibitory effect of SKLB-178 on endothelial cells migration. As indicated in Figure 4A, the number of migrating cells was significantly diminished by SKLB-178 (1 μM) as compared with vehicle. In transwell invasion assay, SKLB-178 strongly inhibited HUVEC invasion at a concentration of 1 μM. The similar effect was also observed in Matrigel-based tube formation assay, in which the ability of HUVEC assembling into branched capillary-like structure was also effectively depressed by 1 μM SKLB-178. The ability of SKLB-178 to inhibit the VEGFR signaling was further assessed in HUVEC. As the Figure 4B shows, VEGFR2 (KDR) and its downstream signaling molecule ERK were activated upon stimulation by VEGF, while this activation could be intensively suppressed by SKLB-178 at concentrations higher than 0.3 μM.

To further test the antiangiogenic effect of SKLB-178 *in vivo*, transgenic zebrafish assay was adopted for assessment. As shown in Figure 4C, SKLB-178 at a concentration of 1 μM could partly inhibit the growth of zebrafish intersegmental blood vessels, and 3 μM SKLB-178 led to almost complete blockade of the intersegmental vessel formation. The antiangiogenic activity of SKLB-178 was comparable to that of the positive control Sorafenib. All of these results revealed that SKLB-178 could efficiently restrain angiogenesis *in vitro* and *in vivo* mainly through suppression of VEGFR signaling pathway.

### Antitumor efficacy and mechanisms of action of SKLB-178 in human NSCLC xenograft models

**Figure 5:**
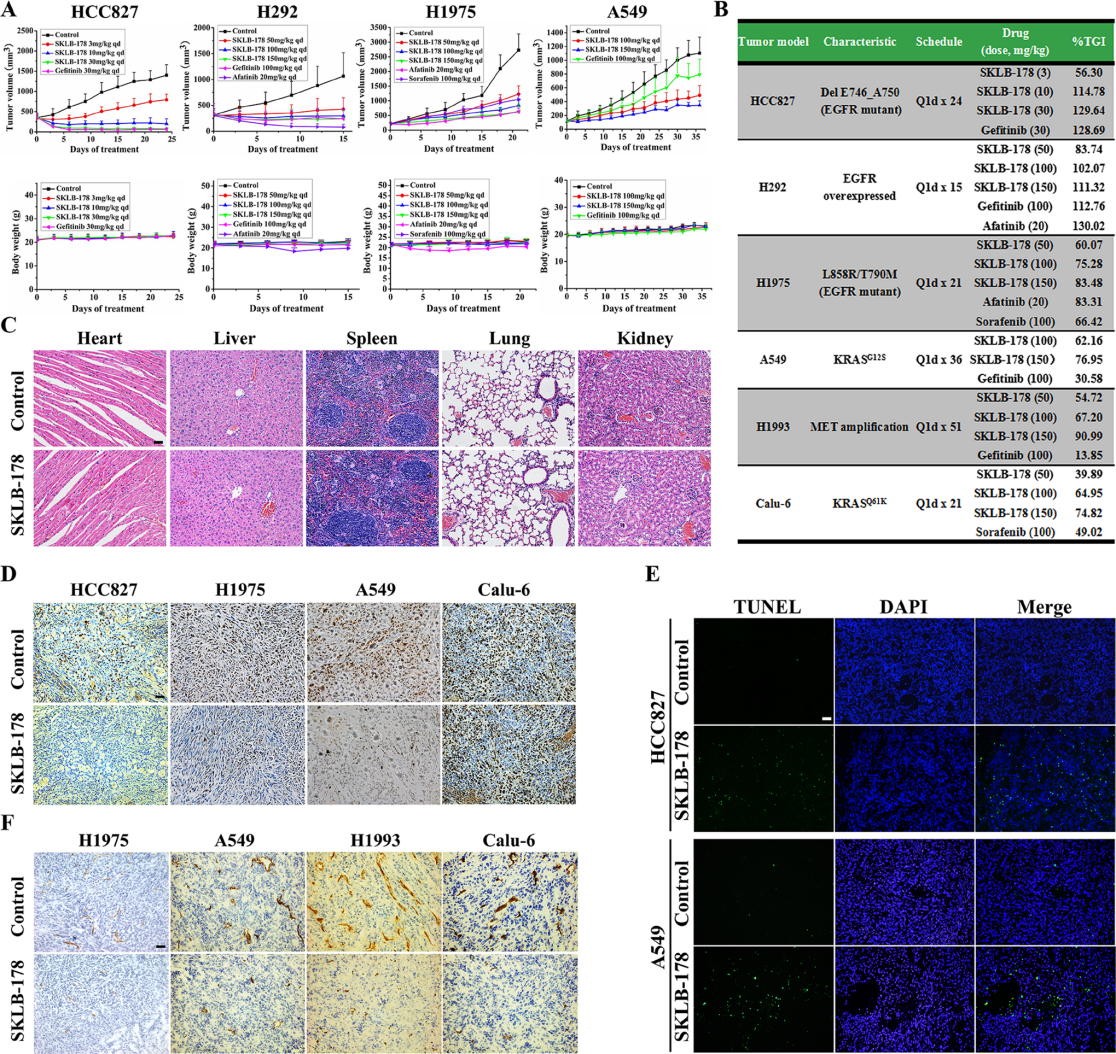
*In vivo* pharmacodynamic studies of SKLB-178 (**A**) Tumor volumes and body weights in HCC827, H292, H1975, and A549 xenograft models. Points, mean tumor volume or body weight; bars, SD. (**B**) Summary of anti-NSCLC activity of SKLB-178 in all xenograft models. (**C**) H&E staining of paraffin sections of the major organs from vehicle control and 150 mg/kg SKLB-178 treatment groups in A549 model. Scale bar, 100 μm. (**D**) Immunohistochemical staining was used to determine the anti-proliferative effect of SKLB-178. Paraffin sections of tumor tissues from 10 mg/kg (just HCC827 model) or 150 mg/kg SKLB-178 treatment groups were used for analysis. Scale bar, 100 μm. (**E**) Induction of apoptosis by SKLB-178 was detected in HCC827 and A549 models. Paraffin sections of tumor tissues from 10 mg/kg (HCC827 model) or 150 mg/kg (A549 model) SKLB-178 treatment groups were used for analysis. Scale bar, 100 μm. (**F**) Immunohistochemical analysis was performed to determine the anti-angiogenesis activity of SKLB-178 *in vivo*. Frozen sections of tumor tissues from vehicle and 150 mg/kg SKLB-178 treatment groups in each model were used for staining. Scale bar, 100 μm.

The *in vivo* anti-tumor efficacy of SKLB-178 was assessed in HCC827, H292, H1975, A549, H1993 and Calu-6 tumor xenograft models. Once daily oral administration of SKLB-178 produced a dose-dependent inhibition of tumor growth in all models examined (Figure 5B). Full growth inhibition profiles are shown for four typical xenograft models (Figure 5A). Among them, HCC827 and H292 models were sensitive to EGFR inhibitor. Both SKLB-178 and Gefitinib could result in tumor regression in these two models at middle and high doses (Figure 5A). In Gefitinib-resistant H1975 model, SKLB-178 was still able to suppress the growth of tumor efficaciously, and the anti-tumor activity of SKLB-178 at the high dose (150 mg/kg) was similar to that obtained with the positive control Afatinib (20 mg/kg). Specifically, TGI values were 83.48% and 83.31%, respectively (Figure 5B). Moreover, Sorafenib, an angiogenesis inhibitor, also exhibited a moderate tumor inhibitory activity in H1975 model, with tumor growth inhibition rate of 66.42% at 100 mg/kg. In the A549 xenograft model, oral administrations of SKLB-178 at 100 and 150 mg/kg inhibited tumor growth in a dose-dependent manner at rates of 62.16% and 76.95%, respectively (Figure 5B). However, treatment with Gefitinib at 100 mg/kg produced only a marginal effect on tumor growth. Of note, when compared with the vehicle group, severe weight loss and pathological changes of major organs were not observed in SKLB-178-treated animals even at a high dose of 150 mg/kg (Figure 5A and 5C), indicating the low toxicity of SKLB-178. By comparison, the weight of mice treated by Afatinib fluctuated remarkably, demonstrating potential side effects of this agent. Collectively, these results showed that SKLB-178 was efficacious and well tolerated in NSCLC xenograft models, and had broader anti-NSCLC activity than Gefitinib.

To elucidate the mechanisms of action of SKLB-178-mediated anti-tumor effects *in vivo*, immunohistochemical staining assays were carried out on the tumor tissues. As indicated in Supplementary Figure S1, SKLB-178 potently inhibited the phosphorylation levels of EGFR and Src just as depicted in *in vitro* assays, leading to a significant decrease in proliferating tumor cells (Ki67-positive cells) and substantial increase in apoptotic cells (TUNEL-positive cells) compared with the corresponding control in HCC827 and A549 models (Figure 5D and 5E). The anti-proliferation effect of SKLB-178 was also detected in H1975 xenograft model, but not in Calu-6 model (Figure 5D). In addition, SKLB-178 also exhibited effective anti-angiogenesis activity *in vivo*, and a significantly reduced microvessel density was observed in the SKLB-178-treated tumors (Figure 5F). Taken together, these results demonstrated that SKLB-178 could still inhibit specific targets *in vivo*, and exert its anti-tumor effects through inhibition of proliferation and induction of apoptosis, as well as restraining angiogenesis.

### Antimetastatic activity of SKLB-178

**Figure 6:**
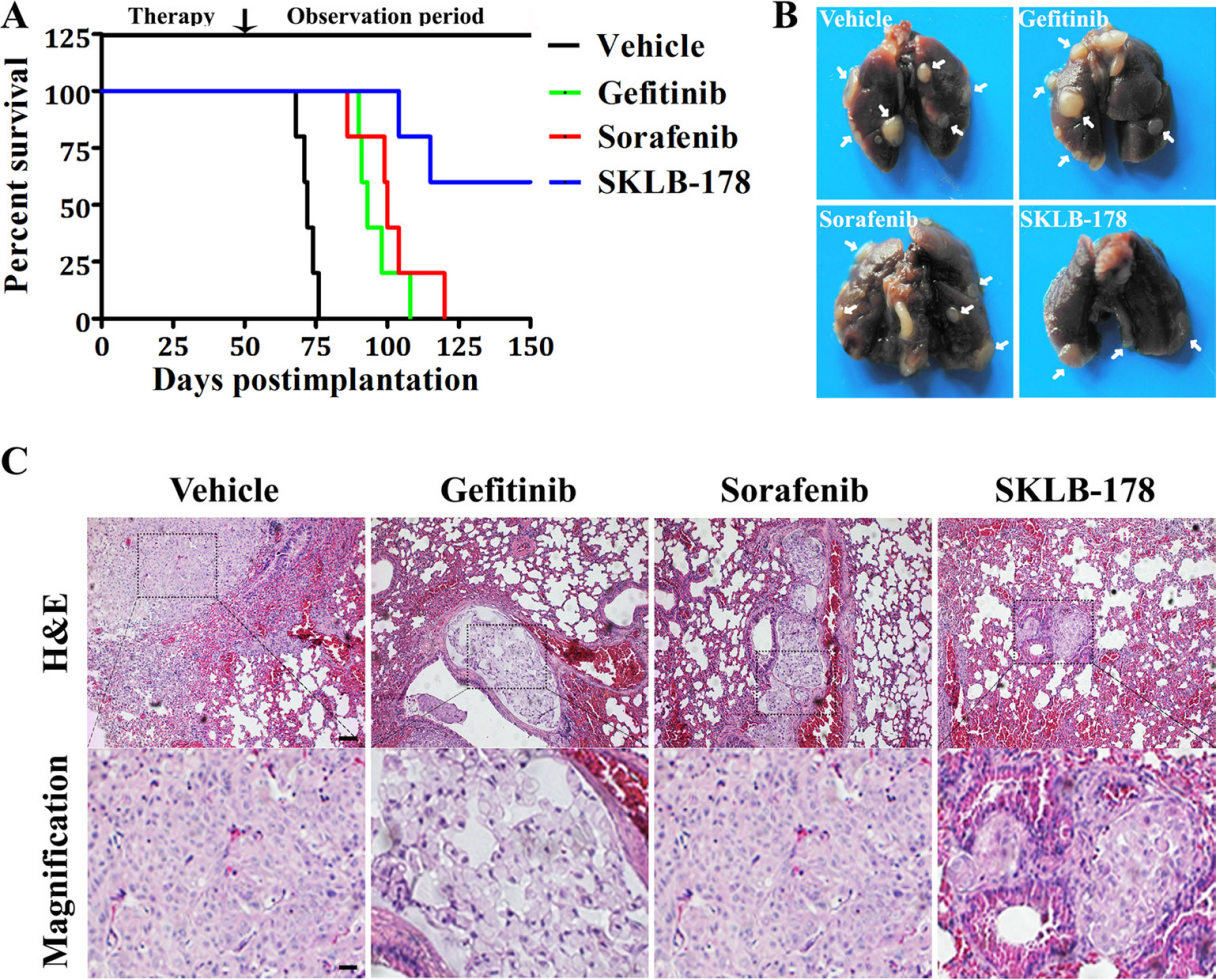
Anti-metastatic activity of SKLB-178 in A549 intravenous implantation model (**A**) Kaplan-Meier survival curves. (**B**) Representative images of intact lungs from dead mice in every group. The white arrows indicate metastatic nodules. (**C**) H&E staining of lungs from mice that died in every treatment group. Scale bars represent 100 μm and 25 μm for low and high magnification, respectively.

We ulteriorly assessed the effect of SKLB-178 on the metastasis-associated survival by injecting A549 cells via tail vein in BALB/c nude mice. As shown in Figure 6A, the mean survival time (MST) was 72.2 days in vehicle treatment group, with all mice expired by day 76. SKLB-178 treatment significantly prolonged survival of animals, and metastasis-related mortality just occurred at day 104 and 115. There were still 3 mice alive even at the end of the experiment (day 150). In contrast, the mean survival time of mice in Gefitinib and Sorafenib treatment group were 96 days and 101.8 days, respectively, indicating a weaker antimetastatic activity compared with SKLB-178. Additionally, histological anatomy of dead animals demonstrated that they all died of lung metastases (marked by white arrow) (Figure 6B). H&E staining further confirmed that the dead mice developed macrometastasis in the lungs (Figure 6C).

## DISCUSSION

The EGFR family, also known as the ErbB/HER family, is a group of receptor tyrosine kinases implicated in the development of cancer, especially NSCLC. Src is known to be both downstream transducer and upstream activator of EGFR. It can be activated by EGFR, and also transactivate EGFR by phosphorylating tyrosine residue Y845 [29, 30]. They have synergistic functions. As many tumors overexpress both EGFR and Src, it is reasonable to infer that inhibition of both targets may be beneficial for cancer therapy. Furthermore, VEGFR and EGFR share common downstream signalings, and have direct and indirect auxo-action on the development of tumor and angiogenesis [11, 31, 32]. Combined inhibition of EGFR and VEGFR signaling pathways has been proven effective in the treatment of NSCLC [11, 33]. Hence, synergistic targeting of these targets mentioned above may be beneficial for NSCLC therapy. SKLB-178 reported in this account is a small molecule multi-kinase inhibitor with inhibitory potency against EGFR, Src, and VEGFR2 kinases.

There are at least two advantages of SKLB-178 in the treatment of NSCLC. The first one is its inhibitory potency against both EGFR-activating mutations and EGFR gatekeeper mutation (T790M). The latter is the main acquired resistance mutation to Gefitinib, and accounts for up to 50% of all resistant cases [7, 34]. Thus, SKLB-178 can effectively restrain the proliferation of both HCC827 (harboring EGFR-activating mutation) and H1975 (harboring EGFR T790M mutation) *in vitro* and *in vivo*. The second one is that SKLB-178 can not only inhibit the growth of tumor cells, but also block tumor angiogenesis. This could be used to interpret why SKLB-178 shows weaker anti-viability activity against H1975 cells compared with the positive control Afatinib *in vitro*, but could achieve comparable anti-tumor potency in H1975 xenograft model. This is also the main reason why SKLB-178 significantly prolongs survival compared with either EGFR inhibitor or angiogenesis inhibitor in the experimental metastasis model.

Another attractive point of SKLB-178 is its anti-proliferation effect in some KRAS mutant cells. KRAS is a most frequently mutated oncogene causing NSCLC patients resistant to Gefitinib. It has been one of the most powerful predictive markers for resistance to EGFR inhibitors [35, 36]. However, due to the complexity of KRAS signaling, there are still many challenges for therapeutic targeting. Recent studies have demonstrated that Src is a potential target for the treatment of KRAS mutant tumors [37, 38], and targeting Src with the pan SFK inhibitor Dasatinib could sensitize some KRAS mutant tumors to Cetuximab [39]. On the basis of the previous studies between KRAS and Src, the potency of SKLB-178 against some KRAS mutant NSCLC cells, especially A549 (KRAS^G12S^), may be mainly attributed to its inhibitory activity against Src kinase.

In summary, SKLB-178 is a compound that combines inhibition of EGFR-activating and resistant mutations, as well as Src and VEGFR2 kinases in one molecule. It effectually inhibited tumor cell growth and angiogenesis both *in vitro* and *in vivo*, exhibiting broad anti-NSCLC activity in preclinical pharmacodynamic study. SKLB-178 has the convenience of oral administration, and is well tolerated. Taken together, SKLB-178 deserves to be further developed as a potential drug candidate for NSCLC therapy.

## MATERIALS AND METHODS

### Compounds and cell lines

SKLB-178 (Figure 1A) was synthesized at the State Key Laboratory of Biotherapy, Sichuan University (details for the synthesis of SKLB-178 see Supporting Material). Gefitinib, Afatinib, Vandetanib, and Sorafenib were obtained from commercial source. All of the compounds were dissolved in dimethyl sulfoxide (DMSO), and the final concentration of DMSO in the incubation mixture did not exceed 0.1% (v/v) in each experiment *in vitro*. Human NSCLC cell lines were obtained from American Type Culture Collection (ATCC, Rockville, MD) and National Platform of Experimental Cell Resources for Sci-Tech (China). All tumor cell lines were cultured according to standard procedures and passaged for less than 6 months after receipt for resuscitation. Human umbilical vein endothelial cell (HUVEC) was isolated from human umbilical cord veins and grown in EGM-2 medium (Millipore, USA). HUVECs at passage 2–6 were used for all studies.

### Kinase inhibition assay

Kinase inhibition assay was conducted by KinaseProfiler service provided by Millipore as described previously [40].

### Cell viability assay

Cell viability was measured using MTT assay. Cells growing in 96-well plates were treated with DMSO control or the indicated compounds for 72 h. 20 μL of MTT solution (5 mg/mL, Sigma, USA) was then added to each well, and the plates were incubated for another 4 h at 37°C. The formazan crystals were dissolved with acidified SDS (20%, w/v) overnight, and absorbance was detected at 570 nm on Multiskan MK3 (Thermo Scientific, USA).

### Colony formation assay

Cells were seeded in six-well plates at a concentration of 5,000 cells per well, and cultured in the presence of agents for 10 days. At the end of incubation, cells were fixed with methanol for 15 min, followed by staining with crystal violet (0.05%, w/v) for another 15 min.

### EdU cell proliferation assay

Cells growing in 96-well plates were cultured in the presence of different concentrations of agents for 24 h. Cell proliferation was determined by EdU incorporation assay according to the manufacturer^’^s protocol (RIBOBIO, China). The images were captured by ArrayScan VTI HCS reader (Thermo Scientific, USA).

### Flow cytometry for cell cycle and apoptosis analysis

Cells were treated with vehicle or agents for 24 h and harvested. For cell cycle analysis, cells were incubated with 50 μg/mL propidium iodide, 100 μg/mL RNase and 0.1% Triton X-100 for 30 min in the dark. The cell cycle profiles were determined by flow cytometry (Becton Dicknson, USA) and analyzed using the ModFit LT 3.2 software (Verity Software House, USA). For apoptosis analysis, cells were detremined using the Annexin V-FITC/PI apoptosis detection kit (KeyGEN Bio TECH, China) following the manufacturer^’^s instruction.

### Wound healing assay

Cells were cultured to confluence in 24-well plates and wounded by scratching with a 200-μL sterilized pipette tip, followed by treatment with vehicle or 1 μM SKLB-178 for 20 h. Pictures were taken under an OLYMPUS light microscope.

### Transwell invasion assay

The Transwell compartments (Millipore, USA) were inserted into a 24-well plate and pre-coated with 50 μL diluted Matrigel (BD Biosciences, USA). HUVECs were suspended in EBM-2 medium and seeded in the upper chamber, followed by treatment with vehicle or 1 μM SKLB-178. EGM-2 medium (EBM-2 medium supplemented with various growth factors) was added to the lower chamber, and then cells were allowed to migrate for 24 h at 37°C. The migrated cells were stained with 0.05% crystal violet (w/v) and photographed using a light microscope (Leica, Germany).

### Tube formation assay

The tube formation assay was conducted as described previously [41]. In Brief, 50 μL per well of Matrigel (BD Biosciences, USA) was added to the 96-well plate and allowed to solidify at 37°C for 30 min. HUVECs were then seeded on the Matrigel and cultured in the presence of vehicle or SKLB-178 (1 μM). Cells were photographed with an OLYMPUS digital camera 6 h later.

### Zebrafish

Anti-angiogenesis effect of SKLB-178 and Sorafenib was assessed in transgenic zebrafish (FLK-1: EGFP) following the protocol reported previously [42]. Briefly, zebrafish embryos were incubated with vehicle, SKLB-178 or Sorafenib from 15 h postfertilization (hpf) until 31 hpf. Then zebrafish were anesthetized and imaged under a fluorescence microscope (Carl Zeiss Microimaging, Germany).

### Western blotting

Cells were lysed in RIPA buffer (Beyotime, China) containing protease inhibitor cocktail. Cell extracts were subjected to SDS-PAGE, and then transferred to polyvinylidene fluoride (PVDF) membranes (Millipore, USA), followed by immunoblot with antibodies. All primary antibodies were purchased from Cell Signaling Technology and used at a 1:1,000 dilution, while the horseradish peroxidase-coupled secondary antibodies (Zhong Shan Golden Bridge Bio-technology, China) were used at 1:5,000. Specific proteins were detected using the enhanced chemiluminescene system (Millipore, USA).

### Xenograft studies

All animal studies carried out were approved by the Animal Care and Use Committee of Sichuan University (Chengdu, Sichuan, China). Tumor xenograft models were established by subcutaneously injecting 5 × 10^6^ tumor cells into the hind flank region of BALB/c nude mice. After tumor development, mice were randomized (*n* = 6 each), and drugs were administered orally once daily. Tween-80 (0.1%, v/v) was used as vehicle. Tumor sizes were measured every 3 days with caliper, and the volume was calculated as length × width^2^ × 0.5. Inhibition rate of tumor growth was calculated using the following formula: 100 × {1 - [(tumor volume_final_ - tumor volume_initial_) for the compound-treated group] / [(tumor volume_final_ -tumor volume_initial_) for the vehicle- treated group]}.

For the metastasis study, A549 cells (1 × 10^6^) were injected into female BALB/c nude mice via tail vein, and the animals were immediately divided into four groups: 0.1% tween-80 as vehicle control, 100 mg/kg SKLB-178 treatment group, 100 mg/kg Gefitinib treatment group, and 100 mg/kg Sorafenib group. Drugs and vehicle were administrated orally once a day for 50 days. Survival was determined by observation when the animals became moribund.

### H&E and immunohistochemistry

Tumor tissues and organs were dissected from the sacrificed BALB/c athymic mice after treatment with SKLB-178 for the indicated time. Paraffin-embedded sections of the major organs and tumors were stained with H&E according to the standard protocols. Frozen or paraffin-embedded tumors were subjected to immunostaining with p-EGFR, p-Src, Ki67, TUNEL, or CD31 antibodies from Cell Signaling Technology or Abcam. The representative images were taken under a light microscope (Leica, Germany).

### Statistical analysis

GraphPad Prism v5.0 software was used for statistical analysis. Data were analyzed by the Student *t* test and ANOVA. *P* < 0.05 was considered statistically significant.

## SUPPLEMENTARY MATERIALS


