# Supplementary material for: Preclinical pharmacodynamic evaluation of drug candidate SKLB-178 in the treatment of non-small cell lung cancer
Source: Oncotarget. 2017 Jan 11;8(8):12843–54. doi: 10.18632/oncotarget.14597 (PMC5355060; doi:10.18632/oncotarget.14597)
Supplement: Supplementary file 1 [file oncotarget-08-12843-s001.pdf]

Preclinical pharmacodynamic evaluation of drug candidate SKLB-178 in the treatment of non-small cell lung cancer

Supplementary Materials

SUPPLEMENTARY METHODS

Synthesis of SKLB-178

Commercially available 4-fluoronitrobenzene(1) reacted with 1-methylpiperazine in the presence of K_2CO_3 in DMSO to produce the intermediate 2. The catalytic hydrogenation of the nitro functionality in 2 with palladium on carbon (Pd/C) provided the desired aniline 3 in excellent yield. Reaction of the commercially available 2,4-dichloro-5-nitro-pyrimidine (4) with cyclopentylamineproduced the intermediate 5. Nucleophilic aromatic substitution of the 2-chloro position in 5 with 3 in n-butanol at 90°C yielded the compound 6. Subsequent hydrogenation of the nitro group in 6 using Pd/C as a catalyst provided a good yield of the desired intermediate 7, which was treated with 1-bromo-4-isothiocyanatobenzeneto produce the compound SKLB-178. Yield: (48%, 95% HPLCpurity). 1HNMR (400 MHz, DMSO-d_6): δ 9.22 (s, 1H), 9.09 (s, 1H), 8.39 (s, 1H), 7.83 (d, J = 9.2 Hz, 2H), 7.66 (d, J = 8.0 Hz, 2H), 7.51 (d, J = 8.0 Hz, 2H), 6.93 (d, J = 8.0 Hz, 2H), 4.98-4.94 (m, 1H), 3.15 (m, 4H), 2.75 (m, 4H), 2.46 (s, 3H), 2.05 (m, 4H), 1.68 (m, 2H), 1.23(m, 2H); MS (ESI, positive ion) m/z: 547.1 [M + H]+.

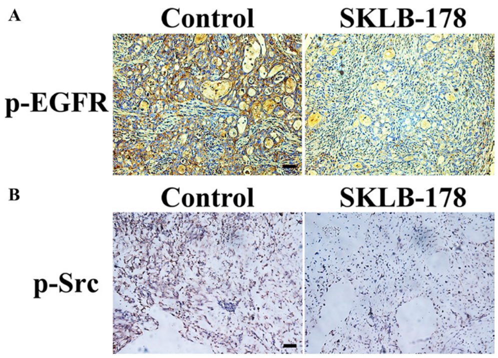

Supplementary Figure S1: SKLB-178 potently inhibited the activities of EGFR and Src *in vivo*. (**A**) Inhibition of EGFR phosphorylation by SKLB-178 was detected in HCC827 xenograft model. Paraffin sections of tumor tissues from vehicle and 10 mg kg SKLB-178 treatmentgroups were used for staining. Scale bar, 100 µm. (**B**) Suppression of Src phosphorylation by SKLB-178 was determined in A549 model. Paraffin sections of tumor tissues from vehicle and 150 mg/kg SKLB-178 treatmentgroups were used for analysis. Scale bar, 100 µm.

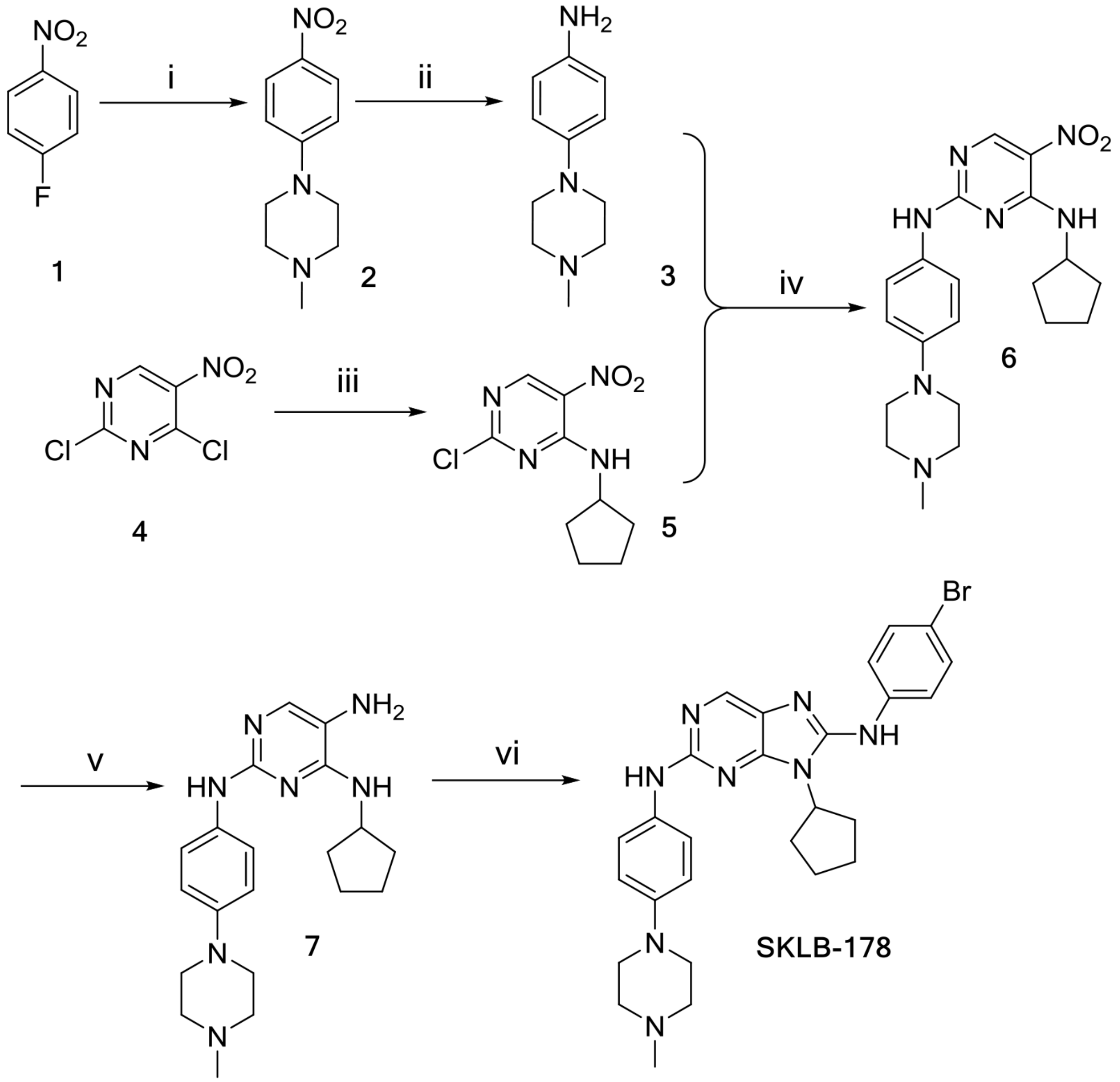

Supplementary Scheme S1: Synthesis of the compound SKLB-178.Reagents and conditions. (**i**)1-methylpiperazine, K_2CO_3, DMSO, rt, 6 h, 95%; (**ii**) H2, 10% Pd/C, EtOH, rt,9 h, 90%; (**iii**) CH^2Cl^2, DIEA, amine, –60°C to room temperature, 60%; (**iv**) n-butanol, 90°C, 5 h, 76%; (**v**) H_2, 10% Pd/C, MeOH, 50°C, 6 h, 85%; (**vi**) CH^2Cl^2, DIEA, EDIC, 1-bromo-4-isothiocyanatobenzene, reflux,12 h, 48%.

Supplementary Table S1: ***In vitro*** **kinase inhibition profile of SKLB-178**

Kinases	Inhibition rate @ 1 µM	Inhibition rate @ 10 µM
EGFR (h)	101	102
EGFR(L858R)(h)	100	100
EGFR(T790M)(L858R)(h)	99	103
ErbB4 (h)	100	—
FAK(h)	7	10
FGFR1(h)	0	0
IGF-1R (h)	11	—
KDR(h)	103	100
MEK(h)	0	1
MET (h)	0	88
MKK4(m)	—	0
mTOR (h)	—	0
PDGFRα(h)	58	—
PDGFRβ(h)	26	27
PI3 Kinase (p110/p85)(h)	6	2
PLK1 (h)	—	0
Src(1-530)(h)	100	101
Syk (h)	28	—
